# Molecular and cellular regulation of thermogenic fat

**DOI:** 10.3389/fendo.2023.1215772

**Published:** 2023-07-03

**Authors:** Cuihua Wang, Xianju Wang, Wenxiang Hu

**Affiliations:** ^1^ GMU-GIBH Joint School of Life Sciences, The Guangdong-Hong Kong-Macau Joint Laboratory for Cell Fate Regulation and Diseases, Guangzhou Laboratory, Guangzhou Medical University, Guangzhou, China; ^2^ Zhongshan School of Medicine, Sun Yat-Sen University, Guangdong, China

**Keywords:** thermogenic fat, energy expenditure, transcription factor, epigenetic modification, intercellular regulation, inter-organ crosstalk

## Abstract

Thermogenic fat, consisting of brown and beige adipocytes, dissipates energy in the form of heat, in contrast to the characteristics of white adipocytes that store energy. Increasing energy expenditure by activating brown adipocytes or inducing beige adipocytes is a potential therapeutic strategy for treating obesity and type 2 diabetes. Thus, a better understanding of the underlying mechanisms of thermogenesis provides novel therapeutic interventions for metabolic diseases. In this review, we summarize the recent advances in the molecular regulation of thermogenesis, focusing on transcription factors, epigenetic regulators, metabolites, and non-coding RNAs. We further discuss the intercellular and inter-organ crosstalk that regulate thermogenesis, considering the heterogeneity and complex tissue microenvironment of thermogenic fat.

## Introduction

Obesity is a chronic and complex condition resulting from an imbalance of excessive energy intake and insufficient energy expenditure, and it is tightly associated with type 2 diabetes, cardiovascular disease, nonalcoholic fatty liver disease (NAFLD), and other metabolic diseases (1). Adipose tissue is a metabolically active organ with significant roles in regulating whole-body energy homeostasis, whose dysfunction causes obesity and related metabolic disorders. Mammals have been shown to possess two classes of fat cells—white and thermogenic adipocytes. White adipocyte contains a large lipid droplet and a few mitochondria and plays an essential role in energy storage in triglycerides. In contrast, thermogenic adipocytes possess multilocular lipid droplets and higher amounts of mitochondria and dissipate energy in the form of heat.

Thermogenic adipocytes consist of brown adipocytes and beige adipocytes. Brown adipocytes are characterized by marker gene *uncoupling protein 1* (*Ucp1*), which uncouples oxidative respiration from ATP synthesis, resulting in energy dissipation as heat (2). The brown adipose tissue (BAT) is predominantly located in the interscapular region of infants and rodents. UCP1-positive multilocular adipocytes were also found in cervical and supraclavicular regions in human adults using positron-emission tomography and computed tomography (PET/CT) imaging (3, 4). Importantly, BAT activity is inversely correlated with body mass index (BMI) and age in humans (5, 6). Moreover, *Ucp1*-deficient mice gain more weight than wild-type mice under thermoneutral conditions (7, 8), while transplantation of mouse BAT or CRISPR-enhanced human or mouse brown-like adipocytes improves glucose tolerance and insulin sensitivity in recipient mice (9, 10). These data suggest the importance of BAT in regulating energy metabolism and homeostasis both in mice and humans. In regard to beige adipocytes, they are predominantly spread in inguinal white adipose tissue (iWAT), and induced in response to cold environment, exercise training or activation of β-adrenergic receptors (β-AR) in mice (11). Intriguingly, the gene profile of mouse beige adipocyte is very similar to that of human BAT in the supraclavicular region during cold exposure (12). Induction of browning in iWAT by transgenic expression of PR domain-containing 16 (Prdm16) increases *Ucp1* mRNA level and protects the mice from diet-induced obesity (13). Therefore, inducing the formation of beige adipocytes may serve as an alternative therapeutic strategy for combating obesity and metabolic diseases.

In this review, we summarize the cell autonomous and non-cell autonomous regulation of the biogenesis and function of thermogenic fat, which will facilitate the development of new therapies for metabolic diseases.

## Molecular regulations of thermogenesis of brown and beige adipocytes

**Figure 1:**
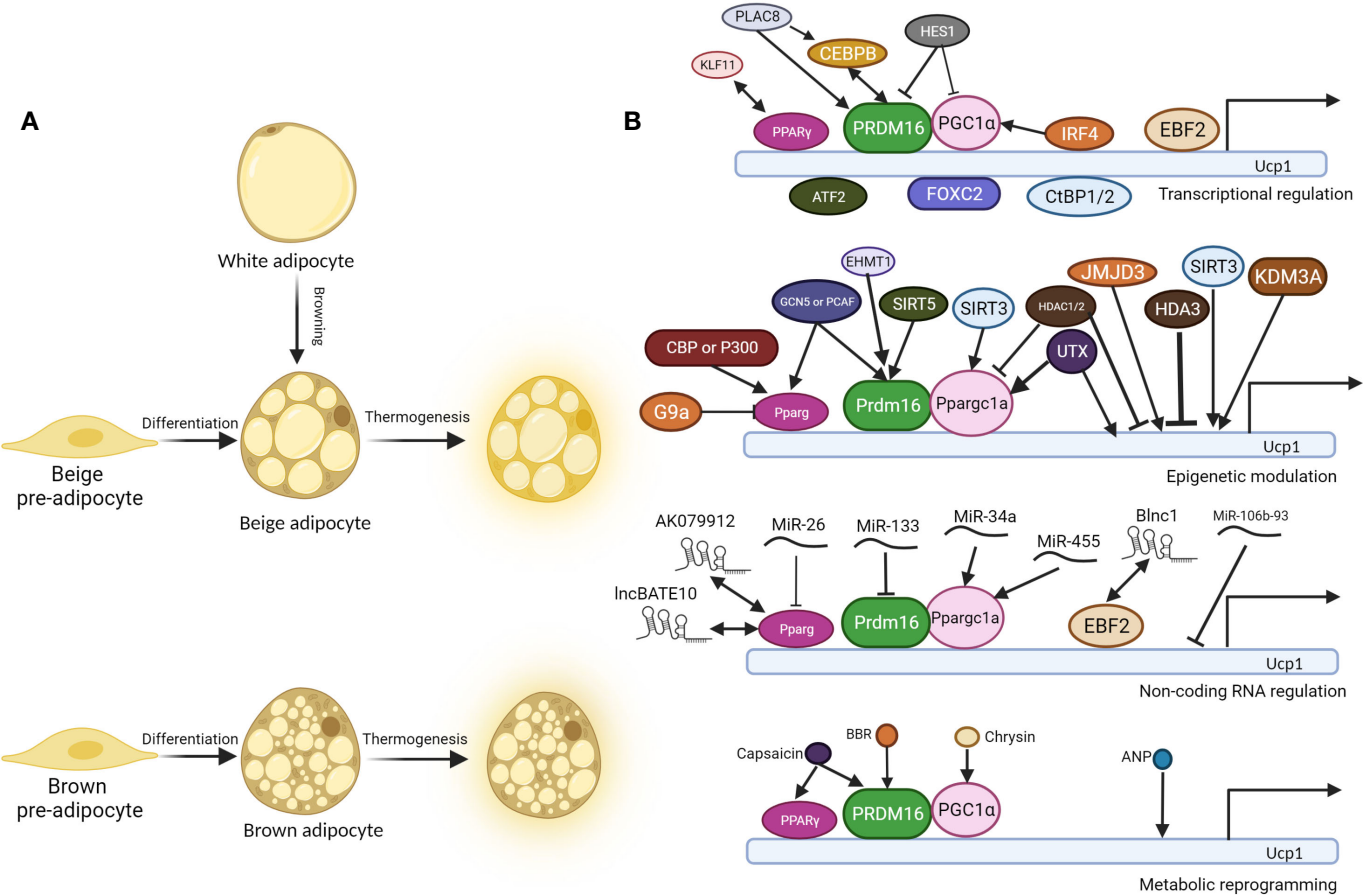
Molecular regulation of thermogenesis of brown and beige adipocytes. **(A)**. Beige pre-adipocyte and brown pre-adipocyte differentiate into beige adipocyte and brown adipocyte respectively. In specific conditions, white adipocytes convert into beige adipocytes, a process called “browning”. Under cold exposure or other signal induction, differentiated brown and beige adipocytes undergo thermogenesis, accompanied by higher glucose and fatty acid uptake, *UCP1* expression, and uncoupled respiration. **(B)**. Regulatory mechanisms behind thermogenesis of brown and beige adipocytes including the following 4 parts: 1. Transcriptional regulation; 2. Epigenetic modulation; 3. Non-coding RNA regulation; 4. Metabolic reprogramming. *UCP1* is one of the most critical thermogenic genes, and its expression is critical for uncoupled cellular respiration. There are three core regulators in the thermogenesis program regulation: PPARγ, PRDM16, and PGC1α, and most other regulators regulate thermogenesis through them. Double-headed arrows indicate protein interaction and complex formation, while arrow-headed and bar-headed lines show inducing and inhibiting effects.

Brown adipocyte and beige adipocyte share similar functions in energy expenditure and thermogenesis, and various molecular events involve in the cell fate determination of thermogenic fat and thermogenesis, including transcriptional regulation, epigenetic modulation, non-coding RNA regulation and metabolic reprogramming (**Figure 1**).

### Transcriptional regulation of thermogenesis in brown and beige adipocytes

The cell fate determination of thermogenic fat is regulated by various adipocyte-specific lineage-determining transcription factors and co-factors as shown in **Table 1**. There are three core regulators in the regulation of thermogenesis of beige and brown adipocyte, proliferator-activated receptor γ(PPARγ), PRDM16 and peroxisome proliferator-activated receptor γ coactivator 1 α (PGC1α). PPARγ was indispensable for the function of both white and brown adipocytes. PPARγ ligands induce the browning of white adipocytes with the cooperation of PRDM16 (30). PRDM16 is highly expressed in brown adipocyte cells, and overexpression of PRDM16 leads to the browning of white adipocytes. Consistently, knock down of PRDM16 causes to the loss of brown fat cell identity (32). PGC1α also plays essential roles in energy metabolism and homeostasis. Although mice without PGC1α underwent normal brown fat differentiation, it accompanied with decreased thermogenic genes induction (25).

**Table 1:** Transcription regulators behind thermogenesis of brown and beige adipocytes.

Factors	Type	Model system	Function	Ref.
**ATF2**	TF (+)	Interscapular BAT (IBAT)	Phosphorylated form promotes UCP1 expression	(14)
**C/EBPα**	TF (+)	3T3-L1 preadipocytes	Inhibits the expression of white fat genes and promotes the expression of brown-specific genes	(15)
**C/EBPβ**	TF (+)	Skin fibroblasts from mouse and man	Form complex with PRDM16 to switch myoblastic precursors to brown fat cells	(16)
**CtBP1/2**	Coregulator (+)	3T3-L1 adipocytes	Interacts with C/EBPα to inhibit the expression of white adipocyte genes	(15)
**EBF2 (COE2)**	TF (+)	Primary brown and white preadipocytes	Recruits PPARγ to BAT specific genes	(17)
**FoxC2**	TF (+)	Transgenic mice with FoxC2 overexpression in fat	Transcription activates UCP1	(18)
**HES1**	TF (-)	Mouse model	Binds promoters of *Prdm16* and *Ppargc1a* to inhibit their expression	(19)
**IRF4**	TF (+)	Mouse model	Interacts with PGC1α to drive *Ucp1* expression	(20)
**IRX3, IRX5**	TF (-)	Primary human adipose–derived progenitor cell cultures	Knockdown of IRX3 or IRX5 restore thermogenesis induced by risk allele	(21, 22)
**KLF11**	TF (+)	hMADS-3 cells were differentiated into mature adipocytes	Cooperates with PPARγ to activate and maintain brite selective gene program	(23)
**MRTFA**	TF (-)	White adipose tissue from MRTFA(-/-) mice	Under the control of BMP7-ROCK signaling axis and inhibits brown-selective genes’ expression in white adipose tissue	(24)
**PGC1α**	Coregulator (+)	Immortal preadipocyte lines from mice lacking PGC1α	Plays essential roles in brown fat thermogenesis	(25–28)
**PLAC8**	Coregulator (+)	Brown preadipocyte lines	Induces the expression of *C/EBPβ* and *Prdm16*	(29)
**PPARγ**	TF* (+)	White adipocytes and mouse model	Acts collaboratively with PRDM16 to induce brown fat gene program	(30, 31)
**PRDM16**	Coregulator (+)	Brown fat precursors, white fat cell progenitors and white fat depots	Activates expression of PGC1α, UCP1 and Dio2	(30–33)
**PRDM3**	Coregulator (+)	Mouse model with PRDM16/PRDM3 double-knockout	Reduces BAT specific genes’ expression in the knockout mice	(34)
**Rb and p107**	TF (-)	p107-/- mice and adult primary preadipocytes	Repress the expression of PGC1α and UCP1	(35)
**RIP140**	Coregulator (-)	3T3-L1 adipocytes, RIP140-null mice	Suppresses adipocyte oxidative metabolism and mitochondrial biogenesis	(36–38)
**SIRT1**	Coregulator (+)	3T3-L1 cells and mouse model	Catalyzes deacetylation of PPAR γ Lys268 and Lys293, and recruits PRDM16 to Pparγ, to induce BAT genes	(31)
**SMAD3**	TF (-)	Smad3-deficient mice	Represses PGC1α expression	(39)
**SRC1**	TF (+)	SRC-1-/- mice	Reduces energy expenditure	(40)
**TBX15**	TF (+)	Adipose tissue in 129/Sv mouse pups	Induces expression of brown phenotypic marker genes	(41)
**TFAM**	mitochondrial TF (+)	TFAM floxed (TFAMf/f) mice	Knocking down TFAM decreases mtDNA copy number and Complex I activity	(42)
**TIF2**	TF (-)	TIF2-/- mice	Enhances adaptive thermogenesis in the KO mice	(40)
**TLE3**	TF (-)	Brown Preadipocytes, mice lacking TLE3	Disrupts interaction between Prdm16 and PPARγ, and suppresses brown-selective genes	(43)
**TWIST1**	TF (-)	Mouse model	Interacts with PGC1α to suppress brown thermogenesis gene	(44)
**USF1**	TF (-)	Mice lacking Usf1	Increases BAT-facilitated thermogenesis in the *Usf1* knockout mice	(45)
**ZFP423**	TF (+)	3T3-L1, 3T3 and Zfp423 knockout mice	Activates *Pparg* expression and increases adipocyte differentiation	(46–48)
**ZFP516**	TF (+)	Zfp516 knockout embryos	Activates UCP1 and PGC1α, to promote a BAT program	(49)

As will discussed in more details below, there are more than 30 transcriptional regulators identified to positively or negatively regulate the formation and function of beige and brown adipocytes, and most of them function through the above three core regulators. CCAAT enhancer-binding protein beta (C/EBPβ) forms a transcriptional complex with PRDM16 to induce brown fat cell determination and differentiation (16). In contrast, CCAAT enhancer-binding protein alpha (C/EBPα) acts collaboratively with other corepressors C-terminal-binding protein 1/2 (CtBP1/2) to repress the expression of white fat genes (15). Early B-cell factor 2 (EBF2), a selective marker of brown and beige precursors (50), regulates the cell fate determination of brown fat precursor cells and the expression of thermogenic genes (17). Brown adipocytes isolated from mice with *Ebf2* deficiency exhibit diminished mitochondrial density and larger lipid droplets (51). Interferon regulatory factor 4 (IRF4), which is induced by cold and cAMP, interacts with PGC1α to promote the expression of PRDM16 and then drive the expression of thermogenic genes (20). Claussnitzer et al. found that rs1421085 T-to-C single-nucleotide variant disrupts the function of AT-rich interative domain-containing protein 5B (ARID5B) that repress the expression of Iroquois homeobox protein 3 (IRX3) and Iroquois homeobox protein 5 (IRX5), which further result in a shift from beige adipocytes to white adipocytes (21). Loft et al. reported that kruepple-like factor 11 (KLF11), which is induced by PPARγ agonists, acts in cooperation with PPARγ to activate beige-selective gene program (23). Zinc finger transcription factors also play important roles in thermogenesis. Gupta et al. reported that zinc finger protein 423 (Zfp423) expression is enriched in white adipocytes compared to brown adipocytes and is repressed upon cold exposure (46). Zfp423 inhibits the activity of EBF2 and suppress PRDM16 activation to maintain white adipocyte identity, and loss of adipocyte Zfp423 induces an EBF2 NuRD-to-BAF coregulator switch and promotes thermogenic genes (47). Dempersmier et al. stated that zinc finger protein 516 (Zfp516) directly binds to the proximal region of the *Ucp1* promoter and activates its expression to induce white fat cell browning and the development of brown fat cells (49). Taken together, the formation and function of thermogenic fat greatly rely on a complex transcriptional network coordinated by a set of core transcriptional factors.

### Epigenetic modulation behind thermogenesis of brown and beige adipocytes

Adipogenesis is involved with complicated epigenetic remodeling that mainly include histone modification and DNA methylation, the two fundamental processes that play crucial roles in the regulation of gene expression and genome stability. In general, Histone modifications modulate chromatin structure, influencing gene accessibility and transcriptional activity, while DNA methylation directly modifies the DNA sequence, leading to gene silencing. A lot of studies have demonstrated the roles of epigenetic modulators in regulating the formation and function of thermogenic adipocytes (**Table 2**). In this review, we specifically focused on the role of histone modification, including histone acetylation, histone deacetylation, histone methylation, and histone demethylation.

**Table 2:** Epigenetic regulators behind thermogenesis of brown and beige adipocytes.

Histone modification	Epigenetic regulators	Influenced gene	Roles	Ref.
**Histone acetylation**	CBP and P300	*Pparγ*	Promotes adipocyte differentiation	(52)
	GCN5 and PCAF	*Pparγ* and *Prdm16*	Facilitates brown adipogenesis	(53)
**Histone deacetylation**	HDAC1 and HDAC2	*Ucp1* and *Pgc1α*	Negatively regulates thermogenic program in brown adipocytes	(54)
	HDAC3	*Pparg*, *Ucp1* and *Ppara*	Inhibits WAT browning	(55)
	HDAC9	*C/EBPα*	Negative regulates adipogenic differentiation	(56)
	HDAC11	*Brd2*	Suppresses brown adipocyte differentiation	(57)
	SIRT1	*Pparγ*, *sFRP1*, *sFRP2*, and *Dact1*	Induces browning of WAT and enhances BAT function	(31, 58, 59)
	SIRT2	*Foxo1* and *Pparγ*	Suppresses adipocyte differentiation	(60)
	SIRT3	*CREB* and *PGC1α*	Activates mitochondria functions and adaptive thermogenesis in brown adipose	(61)
	SIRT5	*Pparγ* and *Prdm16*	Promotes subcutaneous white adipose tissue browning	(62)
	TET	*Ucp1* and *Pgc1α*	Inhibits thermogenic genes’ expression	(63)
**Histone** **Methylation**	MLL3	*aP2*	Promotes brown and white adipocytes differentiation	(64)
	MLL4	*C/EBP*s and *Pparγ*	Promotes brown and white adipocytes differentiation	(65, 66)
	EHMT1	*Prdm16*	Promotes BAT-mediated adaptive thermogenesis	(67)
	G9A	*Pparγ*	Inhibits brown and white adipocytes differentiation	(68)
	KMT5c	*Trp53*	Activates thermogenic program in adipocytes	(69)
	DOT1L	*Ucp1* and *Prdm16*	Inhibits thermogenic adipocyte differentiation and function	(70)
**Histone Demethylation**	LSD1	*Ppara*	Promotes white adipocyte browning	(71)
	LSD2	Brown adipogenesis genes, such as *Ucp1*	Promotes brown adipocyte differentiation	(72)
	KDM5A	*C/EBPβ* and *Wnt6*	Promotes preadipocyte differentiation	(73)
	Kdm3a	*Ppara* and *Ucp1*	Promotes white adipocyte browning	(74–76)
	Jmjd3	*Rreb1*, *Ucp1* and *Cidea*	Promotes browning of WAT	(77)
	UTX	*Ucp1* and *PGC1α*	Regulates brown adipocyte thermogenic program	(78)

Epigenetic modulators catalyze the formation of active epigenetic markers in the regulatory regions of corresponding genes to positively regulate their expression. CREB binding protein (CBP) and histone acetyltransferase p300 (P300), which catalyze histone acetylation of H3K27, improve the expression of *PPARγ* and then promote adipocyte differentiation and white adipocyte browning (52). General control of amino acid synthesis 5-like 2 (GCN5) and P300/CBP-associated factor (PCAF), which acetylate histone H3K9, also facilitate brown adipogenesis through positively regulating the expression of *Pparγ*and *Prdm16* (53).

In regard to histone deacetylation, epigenetic modulators erase pre-settled active epigenetic marker at the regulatory regions of thermogenic genes to negatively regulate their expression. Histone deacetylases (HDAC1, HDAC2, HDAC3, HDAC9 and HDAC11) exert their influences on thermogenesis through deacetylation of H3K27ac (79). HDAC1 and HDAC2 negatively regulate brown adipocyte thermogenic program through decreasing acetylation of histone H3 lysine 27, an active epigenetic marker, on the promoter regions of *Ucp1* and *Pgc1α* to inhibit their expression (54). Ferrari et al. showed HDAC3 deletion induce WAT browning through increased H3K27ac modification at the enhancer region of *Pparγ* and *Ucp1* (55). However, other study revealed that HDAC3 primes *Ucp1* and the thermogenic transcriptional program to maintain the brown adipose tissue identity through deacetylation of PGC1α by HDAC3 (80). Bagchi et al. reported that HDAC11 suppresses WAT browning through physical association with bromodomain-containing protein 2 (BRD2) (57). Other histone deacetylases, including NAD-dependent protein deacetylases-SIRT1, SIRT2, SIRT3, SIRT5, SIRT6, and SIRT7, catalyze the deacetylation of H3K9ac, and/or H4K16ac (58, 81, 82). Shi et al. found that SIRT3 positively correlated with the expression of *Pgc1α* and *Ucp1*, and SIRT3 activates mitochondria functions and adaptive thermogenesis in brown adipose (61). Shuai et al. found SIRT5 promoted the browning of subcutaneous white adipose tissue through regulating H3K9me2 and H3K9me3 modification at the promoter regions of *Pparγ* and *Prdm16* (62). Moreover, ten-eleven translocation (TET) proteins, oxidize 5-methylcytosines and promote specific DNA demethylation (83), were found to inhibit β3-AR dependent thermogenic genes’ expression and white fat browning through indirectly recruiting histone deacetylases to the promoter regions of concerning genes (63).

Histone methylation exerts essential roles in regulating chromatin functional states and usually includes two types of amino acids modification, lysine methyl-transferation and arginine methyl-transferation. Several studies have linked histone methylation with thermogenesis (79). Euchromatic histone methyltransferase 1 (EHMT1), which could catalyze methylation of histone 3 lysine 9 (H3K9me2 and me3), promotes adaptive thermogenesis through stabilizing PRDM16 protein (67). Lysine methyltransferase 5C (KMT5C), a H4K20 methyltransferase, positively regulates thermogenesis through regulating the expression of *transformation related protein 53* (*Trp53*), a repressor of thermogenic program (69). DOT1-like (DOT1L), a lysine 79 of histone H3 (H3K79) methyltransferase, inhibits thermogenic adipocyte differentiation and function through repressing the expression of brown adipocyte tissue-selective genes (70).

Histone demethylases catalyze histone demethylation that usually correlates with enhanced adipogenesis and white adipocyte browning. LSD1, lysine-specific demethylase 1, increases the content of beige adipocytes in aging inguinal white adipose tissue through activating the expression of *proliferator-activated receptor alpha* (*Pparα*) (71). Similarly, lysine-specific demethylase 2 (LSD2) plays its vital roles primarily at the early stage of brown adipocyte differentiation, and its deletion *in vivo* was accompanied with compromised expression of thermogenic genes (72). Tateishi et al. demonstrated lysine-specific demethylase 3A (KDM3A) positively regulates *Pparα* and *Ucp1* expression, and KDM3A-deficient mice developed obesity and hyperlipidemia (74). Pan et al. revealed that JmjC domain-containing protein 3 (JMJD3) demethylases repressive mark H3K27me3 at the promoter regions of *Ucp1* and *Cell death-inducing DFFA-like effector a* (*Cidea*) in order to activate thermogenic program and induce white adipocyte browning (77). Moreover, UTX, ubiquitously transcribed tetratricopeptide repeat on chromosome X, catalyzes demethylation of H3K27me2/3 at the promoter region of *Ucp1* and *Pgc1α* to positively regulate their expression and promote brown adipocyte thermogenic genes expression (78). Altogether, various epigenetic remodelers act through altering histone acetylation and methylation dynamics to regulate the thermogenic program in response to the external stimuli.

### Non-coding RNAs regulation of thermogenesis of brown and beige adipocytes

Non-coding RNAs, including microRNAs (miRNAs) and long non-coding RNAs (lncRNAs), play important roles in the development and physiology of white, brown and beige adipocytes, and non-coding RNAs themselves can serve as markers of different adipocyte tissue depots (**Table 3**).

**Table 3:** Non-coding RNAs behind thermogenesis of brown and beige adipocytes.

Non-coding RNAs	Regulation	Model system	Roles	Ref
**miR-26**	+	Human multipotent adipose-derived stem (hMADS) cells	Represses activity of ADAM17 to increase white adipocytes browning	(84)
**miR-27**	–	Human adipose-derived stem cells, Male C57BL/6J mice, 3T3-L1 cells …	Suppresses PPARγ and CEBPα, targets prohibitin (PHB) to inhibit adipogenesis, and upregulates UCP1, PRDM16 and PGC1α	(85–90)
**miR-30**	+	Brown preadipocyte cell line, SVFs, and C57BL/6 male mice	Upregulates thermogenic genes’ expression	(91)
**miR-32**	+	WT-1, iWAT SVF cells and C57BL6/J mice	Promotes BAT thermogenesis and WAT browning	(92)
**miR-34a**	–	Male C57BL/6 mice and SVF cells	Suppresses FGF21 and sirtuin1 (SIRT1) and fat browning	(93, 94)
**miR-106b-93**	–	Mouse brown preadipocyte cell line, primary mouse stromal vascular fraction (SVF) cells, and C57BL/6J mice	Knockdown of miR-106b-93 increases brown fat-specific genes’ expression	(95)
**miR-125-5p**	–	C57Bl/6J mice	Inhibits WAT browning	(96)
**miR-133**	–	BAT and SAT to mature brown adipocytes, and mouse model	Impairs *Prdm16*, *Ucp1*, *Pparα* and *Pparγ* expression	(97, 98)
**miR-155**	–	miR-155-/- mice, BAT and igWAT cells isolated from C57BL/6J mice	Targets CEBPβ to impair *Ucp1* and *Pgc1α* expression	(99, 100)
**miR-182 and miR-203**	+	Dgcr8 KO mice and primary brown adipocytes	Knockdown of miR-182 or miR-203 causes reduction of BAT markers	(101)
**miR-193b-365**	+	Primary brown preadipocytes and C2C12 myoblasts	Promotes brown adipocyte adipogenesis by inhibiting *Runx1t1* expression, but its roles were controversial	(102, 103)
**miR-196a**	+	Human WAT-progenitor cells, fat progenitor cells, and C57Bl/6 mice	Suppresses expression of white-fat gene *Hoxc8*	(104)
**miR-328**	+	Mouse model	Inhibition of miR-328 decreases thermogenic genes’ expression	(105)
**miR-378**	+-	C57BL6 mice, and isolated BAT and gonadal WAT	Promotes brown adipogenesis, and inhibits WAT browning	(106)
**miR-455**	+	C3H10T1/2 cells	Activates expression of PPARγ and PGC1α and promotes iWAT browning	(107)
**Blnc1**	+	10T1/2 fibroblasts, 3T3-L1 fibroblasts and mouse model	Form complex with EBF2 to stimulate thermogenic gene program	(108)
**AK079912**	+	Primary SVF cells	Drives thermogenic gene program in white adipocytes	(109)
**LncBATE10**	+	Primary preadipocytes, 3T3-L1 cells and mouse model	Protects PGC1α from degradation	(110)
**NONMMUG024827 lncRNA**	+	Mouse model	Positively regulates adiponectin mRNA levels	(111)
**lncRNA H19**		Mouse model	Binds MBD1 and regulates *Igf2*, *Slc38a4* and *Mest*’s expression	(112)

miRNAs usually exert their functions on regulating thermogenesis through complementary reaction with the UTR regions of mRNA transcripts of effector genes. MiR-26 is upregulated during human adipogenesis and induces brown adipocyte differentiation through directly targeting ADAM metallopeptidase domain 17 (ADAM17) (84). MiR-30b/c target 3’UTR of receptor-interacting protein 140 (RIP140), a negative regulator of thermogenic genes, to promote brown adipose tissue function and the development of beige fat (91). MiR-32 is highly expressed during cold exposure, and increases *fibroblast growth factor 21* (*Fgf21*) expression through repressing the expression of *transducer of ErbB-2.1* (*Tob1*), which further promotes white fat cell browning and BAT thermogenesis (92). Ge et al. showed miR-34a inhibits white adipocytes browning through targeting *fibronectin type III domain-containing protein 5* (*Fndc5*) expression (93), while Fu et al. demonstrated miR-34a promotes the deacetylation of PGC1α and its activation by targeting fibroblast growth factor receptor 1 (FGFR1), klotho beta-like protein (βKL) and NAD-dependent protein deacetylase sirtuin-1 (SIRT1) (94). MiR-106b-93 cluster negatively regulate the expression of *Ucp1* and promote the lipid content in differentiated brown adipocytes (95). Giroud et al. reported miR-125b prevents beige adipocyte formation through decreasing mitochondrial biogenesis (96). miR-133 targets 3’ UTR of *Prdm16* to repress its expression that lead to impaired brown fat differentiation and WAT browning (97, 98). MicroRNA 155 is down-regulated during brown preadipocyte differentiation and inhibition of miR-155 enhances brown adipocyte differentiation and white adipocytes browning. Mechanistically, miR-155 forms a bistable feedback loop with CEBP-β (99). MiR-193b–365, referred to as miR-193b and miR-365, showed two contradictory results, that Sun et al. found that blocking of miR-193b–365 impair brown adipocyte adipogenesis by upregulating the expression of *runt-related transcriptional factor 1 translocation partner 1* (*Runx1t1*) (102), while Feuermann et al. reported that miR-193b–365 are not required for the differentiation and development of BAT (103). The detailed roles of miR-193b–365 *in vivo* and *in vitro* need to be further clarified.

The regulation of lncRNAs in the thermogenesis of brown and beige adipocytes are mainly through interacting with other important transcription factors such as PGC1α, EBF2, and PPARγ (113). Recent study identified Blnc1 as a vital lncRNA in promoting the function of brown and beige adipocytes, and then further experiments demonstrated Blnc1 acts synergistically with EBF2 to drive thermogenic gene program (108). Similarly, lncRNA-AK079912 was also reported to play a positive role in brown preadipocyte differentiation and white adipocytes browning, which is mediated by PPARγ (109). A brown adipose tissue-enriched lncRNA, lncBATE10, was found to be differently regulated in cold or exercise conditions, and it regulates brown adipose tissue gene program through decoying the repressor factor-CUGBP Elav-like family member 1 (CELF1) from *Pgc1α*’s mRNA elements (110). In together, the influences of lncRNAs on the regulatory network of brown and beige adipocytes differentiation remain elusive, and especially their direct roles in affecting core transcriptional factors of thermogenic program need to be further elucidated. In summary, miRNAs and lncRNAs, the tight regulators of gene expression, play an indispensable role in regulating brown and beige adipogenesis, which further complicates the regulatory network of thermogenesis.

### Metabolic reprogramming behind thermogenesis of brown and beige adipocytes

The development and function of thermogenic fat involves intensive metabolic reprogramming (114). **Table 4** summarized the nutrients and metabolites that regulates thermogenesis. Notably, most of the studies were conducted in rodent models and their implications in human need to be further explored.

**Table 4:** Metabolic reprogramming behind thermogenesis of brown and beige adipocytes.

Name	Regulation	Model system	Roles	Ref.
**Atrial Natriuretic Peptide (ANP)**	+	Mouse model	Increases browning of fat cells and upregulates expression of *Ucp1*	(115, 116)
**Berberine**	+	db/db mice	Increases thermogenic genes’ expression	(117)
**Bone Morphogenetic Protein 9 (BMP-9)**	+	Obese mice	Enhances expression of FGF21	(118)
**Capsaicin**	+	TRPV1(-/-) mouse models	Promotes interaction between PPARγ and PRDM16 to induce WAT browning	(119)
**Catecholamine**	+	Mouse model	Binds to β3-AR and promotes white fat browning	(120)
**Chlorogenic Acid**	+	Mouse brown adipocytes and human Adipocytes	Upregulates AMPK expression to enhance PPARγ, PRDM16, and PGC1α expression	(121, 122)
**Chrysin**	+	3T3-L1 cells	Activates AMPK and then upregulates browning proteins’ expression	(123)
**Cinnamicaldehyde**	+	Male C57BL/6J mice	Induces WAT browning and UCP1 expression	(124)
**Curcumin**	+	C57BL/6J mice, and 3T3-L1 and primary white adipocytes	Promotes beige fat cells production and induces white fat browning process	(125–127)
**Ellagic Acid**	+	Rats and hamsters	Upregulates expression of UCP1 and inhibits lipid accumulation	(128, 129)
**Emodin**	+	Obese Mice	Increases expression of beige adipocyte markers	(130)
**Epicatechin**	+	High-fat diet mouse model and cultured human adipocytes	Increases mitochondrial biogenesis-related proteins expression and activates browning of fat cells and WATs	(131)
**Fibroblast Growth Factor 21**	+	*C57BL/6J Fgf21-null and wild-type mice*	Upregulates thermogenic genes expression and regulates PGC1α at post-transcription level	(132, 133)
**Flavan-3-Alcohol**	+	3T3-L1 cells and mice	Increases mRNA expression of UCP1	(134)
**Fucoxanthin**	+	White adipose tissues from mice	Increases β3-AR expression and then stimulates UCP1 expression	(135)
**Glucocorticoids**	–	Murine brown adipocytes	Downregulates UCP1 expression in BATs	(136)
**Irisin**	+	Mouse model	Activates ERK and p38MAPK signalling pathways to induce white fat browning	(137)
**Leptin**	+	Wild type mice and UCP1 deficient mice	Promotes expression of UCP1 and UCP2 in the WATs to reduces white adipose tissue	(138)
**Luteolin**	+	male C57BL/6 mice	Activates browning and thermogenesis	(139)
**Mammalian Target of Rapamycin Complex 1 (mTORC1)**	+	Mouse and human adipocytes, and mice with mTORC1 impairment	Activates browning of fat cells	(140)
**Menthol**	+	Mice and primary white adipocytes	Activates TRPM8 which can upregulate UCP1 and PGC1α expression	(141)
**Neuregulin 4 (NRG4)**	+	Mouse model	Has the potential to promote white fat browning	(142, 143)
**Prostaglandin (PG)**	+	Mouse model	Induces the formation of BAT and white fat browning	(144, 145)
**Purple Sweet Potato (PSP)**	+	Mouse model	Upregulates browning-related genes’ expression	(146)
**Quercetin**	+	Mouse model	Increases brown fat marker genes *Ucp1* and *Elovl3* expression	(147)
**Resveratrol**	+	db/db mice	Promotes lithocholic acid (LCA) in the plasma and faeces	(148)
**Rice Bran**	+	High-fat diet-induced obese mice	Upregulates UCP1 expression and downregulates WAT-specific proteins	(149)
**Sesamol**	+	Mouse system and 3T3-L1 model cells	Inhibits white adipogenic genes and promotes expression of brown fat marker genes	(150, 151)
**Taurine**	+	C3H10T1/2 white adipocytes and mouse model	Induces the browning of WAT	(152)
**Telmisartan**	+	3T3/L1 adipocytes and mouse model	Increases expression of white fat browning key factors	(153, 154)
**3-Hydroxydaidzein**	+	Mouse model	Stimulates the browning of WAT	(155)

Wu et al. reported that NAFLD patients treated with Berberine (BBR) for 1 month exhibited increased brown adipocyte mass and activity in mice, since BBR promotes the DNA demethylation of *Prdm16* promoter to activate its expression (117). Dietary capsaicin induces white adipocyte browning through facilitating the interaction and activation of PPARγ and PRDM16, depending on transient receptor potential vanilloid 1 (TRPV1) channels (119). Chlorogenic acid (CGA), a Chinese traditional medicine, induces brown adipocyte thermogenesis through promoting mitochondria function and glucose uptake (121). Lone et al. and Wang et al. demonstrated that curcumin promotes browning of white adipocytes through upregulating *Ucp1* expression (125, 126). Ellagic Acid (EA), located mainly in fruits and plant extracts, also increases iWAT browning through decreasing the expression of *Zfp423* and *aldehyde dehydrogenase family 1 member a1* (*Aldh1a1*) and increasing thermogenic genes expression (128). Epicatechin (Epi), a cacao flavanol, can induce white adipose tissue browning through improving mitochondrial function and upregulating the expression of key thermogenic genes (131).

Apart from the aforementioned nutrients and small molecules that regulate thermogenesis of brown and beige adipocytes, there are other metabolites performing the similar functions, including flavan-3-Alcohol, fucoxanthin, irisin, leptin, luteolin, Menthol Neuregulin 4 (Nrg4), Prostaglandin (PG), Purple Sweet Potato (PSP), Quercetin, Resveratrol, Rice Bran, Sesamol, Taurine, Telmisartan, and 3-Hydroxydaidzein (134, 135, 137–140, 142–153, 155), which will be discussed in details in the below sections.

## Intercellular communications within thermogenic fat

**Figure 2:**
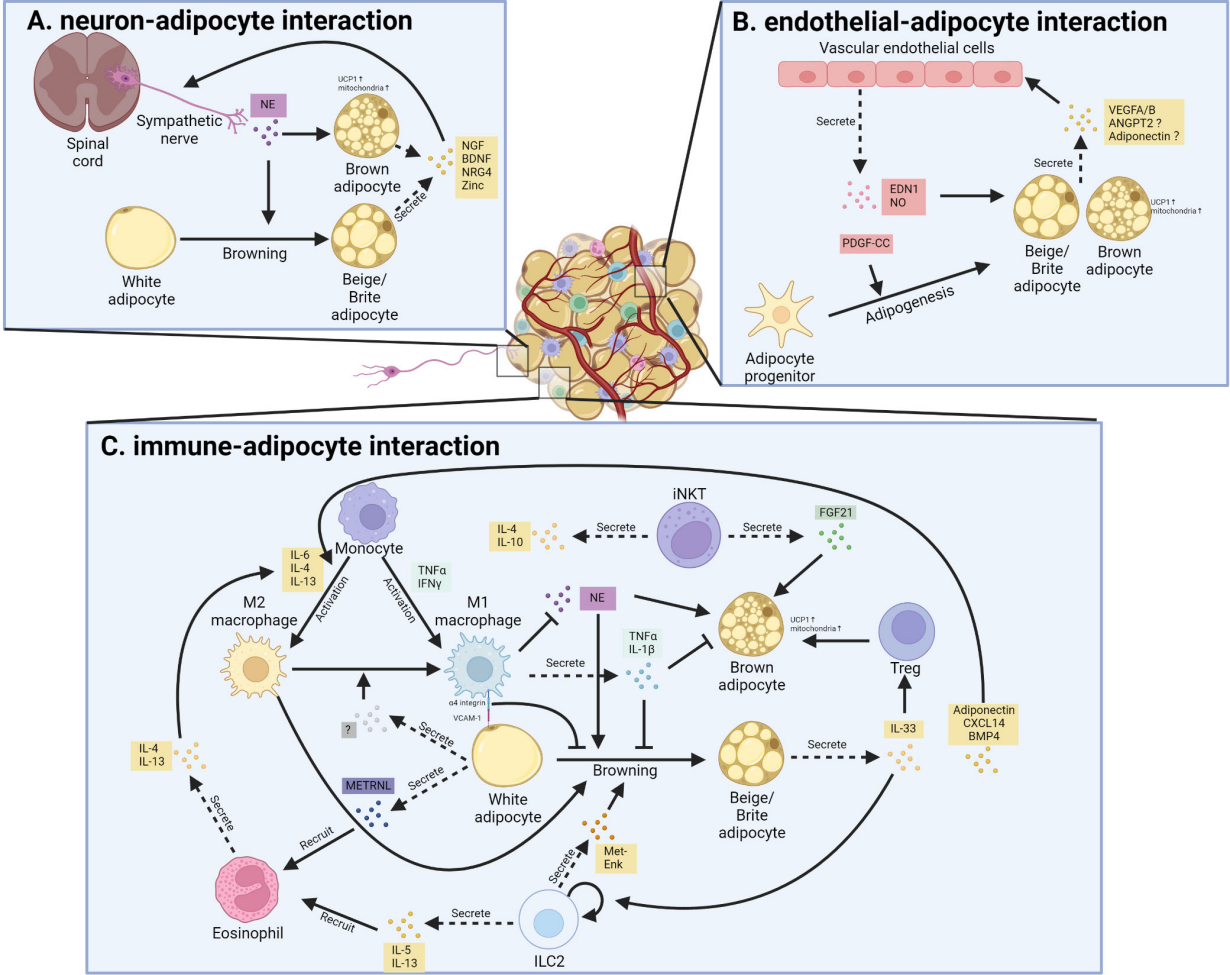
Cellular interaction between thermogenic adipocytes and resident cells. **(A)**. Interaction between sympathetic nerve and thermogenic adipocyte. Sympathetic nerve secretes norepinephrine (NE) that promotes white adipocyte browning and brown adipocyte activation; in turn, beige adipocytes and brown adipocytes promote nerve remodeling through secreting neurotrophic factor, including nerve growth factor (NGF), brain-derived neurotrophic factor (BDNF), neuregulin-4 (NRG4) as well as Zinc. **(B)**. Interaction between vascular endothelial cells and thermogenic adipocyte. Vascular endothelial cells secrete endothelin 1 (EDN1) and nitric oxide (NO) to promote the thermogenic function of brown and beige adipocytes. Besides, the secreted EDN1 and platelet-derived growth factor C (PDGF-C) also regulate the adipogenesis of preadipocytes. Reciprocally, thermogenic adipocytes and their progenitors secrete several factors that promote angiogenesis in adipose tissue. ANGPT2, angiopoietin 2; VEGF, vascular endothelial growth factor. **(C)**. Interaction between resident immune cells and thermogenic adipocytes. Various cytokines and signals mediate the bi-directional communication between thermogenic fat and different kinds of immune cells.

As extensively discussed in a recent review (156), thermogenic fat consists of various cell types or cell states in stromal vascular fractions (SVFs) and mature adipocytes, identified by state-of-art single-cell RNA-sequencing (scRNA-seq) or single nuclei RNA-sequencing (snRNA-seq) in mice (157–165) and humans (157, 162, 165–168). These subpopulations of thermogenic fat, including immune cells, endothelial cells, neurons, smooth muscle cells, Schwann cells, and a few other cell types, create a unique adipose niche and regulate adipose tissue function, such as thermogenic fat turnover, expansion, and remodeling (156). Here we focus on the intercellular crosstalk between thermogenic fat cells and endothelial cells, immune cells, and neurons (**Figure 2**).

### Endothelial cells in the thermogenic adipose tissue

Adipose tissue, especially BAT, is one of the most vascularized tissues in the body (169). A lot of stimuli, including cold, diet, exercise, and nutrition state, modulate angiogenesis and vascular remodeling in adipose tissue. Vascular Endothelial Growth Factor A (VEGFA) and Vascular Endothelial Growth Factor B (VEGFB) are two important angiogenic factors in adipose tissue in response to cold or β3-AR activation. BAT-specific overexpression of VEGFA increases vascularization and improves thermogenesis in mice after cold exposure, and protects mice against diet-induced obesity (170). Similarly, VEGFB promotes the proliferation of endothelial cells and fatty lipid oxidation in thermogenic fat in mice, providing a novel cure strategy for obesity and diabetes diseases (171). Besides, Seki et al. revealed that endothelial-specific *Vegfr2^-/-^* mice showed impaired angiogenesis as well as reduced browning of iWAT, which is modulated through the endothelial cells-derived platelet-derived growth factor-CC (PDGF-CC)-induced signaling pathway, since administration of PDGF-CC upregulated the expression level of *Ucp1* and promoted browning of iWAT both in mice and humans (172). Endothelial cells-secreted endothelin 1 (EDN1) and nitric oxide inhibit biogenesis and the function of brown and beige adipocytes *in vitro* (173, 174). In contrast, endothelial deficiency of lysosomal acid lipase (LAL) impairs vascularization and thermogenesis in BAT and WAT (175). The decreased production of vasodilatory factors and increased vasoconstricting factors production, due to dysfunction of endothelial cells, lead to insulin resistance and diabetes (176). The diverse functions of endothelial cells suggest the existence of different subpopulations. Indeed, Sun et al. observed two distinct types of endothelial cells in human deep-neck BAT using scRNA-seq (162). Vijay et al. also identified three types of endothelial cells in human WAT, with the largest population of endothelial cells defined as fatty-acid-handling microvascular endothelial cells and another subpopulation was lymphatic-derived (167). However, delineating the exact role of each subpopulation of endothelial cells in thermogenic fat needs further investigation. Taken together, these bidirectional communications between thermogenic fat and endothelial cells maintain the adipose homeostasis, and dysfunction of them cause metabolic disorders.

### Immune cells in the thermogenic adipose tissue

Several types of immune cells reside in adipose tissue, including macrophages, natural killer (NK) cells, lymphocytes, dendritic cells, neutrophils, eosinophils, T cells, and mast cells, which play an important role in regulating metabolic homeostasis (177, 178). The adipose immune cells composition is highly variable in response to the nutritional status, as well as environmental stimuli (179).

Among the immune cells that infiltrate into obese adipose tissue, macrophages are functionally and numerically dominant. Activated macrophages are divided into two main categories, M1 macrophages and M2 macrophages. M1 macrophages produce pro-inflammatory cytokines and chemokines, while M2 macrophages secrete anti-inflammatory cytokines that alleviate inflammation. Several studies show that activated M1-like macrophages facilitate the infiltration of other immune cells into obese adipose tissues and impairs insulin sensitivity (180). In detail, studies identified TNFα as a pro-inflammatory cytokine produced from M1 macrophages that suppresses the emergence of thermogenic adipocytes in mice (181). It was also reported that the direct contact between M1 macrophage and white adipocyte could inhibit the browning process as well as *Ucp1* expression in iWAT of mice, mainly though the direct adhesion between α4-integrin in activated M1 macrophage and vascular cell adhesion molecule 1 (Vcam-1) in adipocytes (182). In contrast to M1 macrophages, M2 macrophages exert positive effects on brown adipocyte activity and WAT browning (183). *Signal transducer and activator of transcription 6* (*Stat6*)*-*deficient or macrophage-specific *interleukin-4 receptor α* (*Ilr4α*) knockout mice exhibited impaired BAT thermogenic response, suggesting the positive role of M2 macrophages in BAT thermogenesis, which is further supported by the specific depletion of *Ilr4α* in myeloid cells of mice (184, 185). M2 macrophages could produce catecholamine to sustain adaptive thermogenesis, which may also reflect the situations in WAT browning, as similar recruitment of M2 macrophages were also found in iWAT of cold-induced mice (185, 186). Another study demonstrated that a fraction of M1 macrophages were concentrated around the sympathetic nerve endings in the adipose tissue of obese people (187). Such macrophages are called sympathetic neuron-associated macrophages (SAM), which can transport catecholamine released from sympathetic nerve endings into the cell body and degrade it through monoamine oxidase A, thereby inhibiting the browning of iWAT induced by sympathetic nerve in obese mice (187, 188). Mutually, thermogenic fat could also secrete batokines to regulate the activation and function of macrophages. CXC Motif Chemokine Ligand 14 (CXCL14), one of the batokines secreted by brown adipocytes, promotes the M2 macrophage phenotype in adipose tissue and leads to WAT browning, and *Cxcl14*-deficient mice show impaired BAT activity and altered glucose homeostasis in response to cold exposure (189). Adiponectin is another adipokine that promotes the activation of M2 macrophages and then results in cold-induced browning of WAT in mice (190). Adipose-secreted bone morphogenetic protein 4 (BMP4) also increase the accumulation of M2 macrophages and induce beige fat biogenesis in iWAT of mice (191). Moreover, adipocytes deficient in fatty acid synthase (iAdFASNKO) show increased macrophage polarization, and ablation of macrophage from iWAT in iAdFASNKO mice inhibit beige adipogenesis (161).

Innate lymphoid type 2 cells (ILC2s), another group of adipose resident immune cells, also activate M2 macrophage and regulate thermogenesis in brown and beige adipocytes (192). Activation of ILC2s in the iWAT of mice strongly stimulates the biogenesis of beige fat (193). Mechanistically, ILC2 activation leads to the proliferation of adipocyte precursors and their commitment to the beige fat lineage in mice (193). ILC2 cells also secrete peptide methionine-enkephalin (Met-Enk), which directly targets subcutaneous white adipocytes to induce their browning (194). Moreover, ILC2s respond to the stimulation of interleukin (IL)-33 and produce IL-13 and IL-4 to promote the browning of iWAT in mice, although the cellular origin and signal pathways involved in the endogenous IL-33 production in adipose tissue remain unidentified (193). Consistent with this, *Il-33* deficient mice in iWAT have fewer beige adipocyte formations and larger white adipocyte compared to control mice (194). In a recent study, the unique ILC populations were profiled in human WAT (168), which suggests ILC3s may play a similar role as ILC2 in adipose homeostasis, but function as a more important mediator of adipose tissue inflammation and obesity (168, 194).

Eosinophils are the main IL-4-producing cells in iWAT of mice, and play a key role in the thermogenesis and metabolic homeostasis (195). METRNL, a circulating factor meteorin-like hormone, is induced after exercise and cold exposure in the skeletal muscle and adipose tissue of mice, respectively (196). METRNL promotes alternative activation of adipose tissue macrophages and thermogenic and anti-inflammatory gene programs in iWAT through an eosinophil-dependent increased *Il-4* expression, and blocking IL4/IL13 signaling abrogates METRNL-induced browning of iWAT in mice (196). Moreover, eosinophils-derived IL-4 directly work on PDGFRα^+^ adipocyte precursors to induce beige adipogenesis both *in vitro* and *in vivo* (193). In response to chemokine ligand 11 (CCL11) stimulation, eosinophils are recruited to iWAT and promote type 2 immune responses and beige adipogenesis in mice (197).

### Neurons in the thermogenic adipose tissue

BAT is highly innervated by the complex sympathetic nervous system, which can transmit signals from the central nervous system to BAT (198). BAT thermogenesis is triggered by the release of norepinephrine from its sympathetic nerve terminals, which binds to β3-AR that result in the activation of UCP1 (198). Sympathetic innervation increases after cold exposure in BAT and subcutaneous WAT both in mice and human adults (199). More detailed analysis revealed that sympathetic arborizations in iWAT cover 90% of individual adipocytes, and the sympathetic arborizations are important for the cold-induced browning of iWAT in mice (200). Mutually, the thermogenic fat also regulates the sympathetic innervation and neuron activity. Overexpression of PRDM16 in mice significantly increase the number of sympathetic parenchymal nerve fibers infiltrating the iWAT compared with that in wild-type mice, although the exact mechanism of the recruitment of sympathetic nerves in iWAT remain elusive (200). A recent study revealed that mice lack of fatty acid synthase in fat (iAdFASNKO) activated the sympathetic nerve fiber to result in browning in iWAT of mice (161). Zeng et al. reported that thermogenic adipocytes express mammal-specific endoplasmic reticulum membrane protein (Calsyntenin-3β), which promotes the secretion of S100b from brown adipocytes and stimulates neurite outgrowth in mice (201). Luan group further demonstrated that thermogenic adipocytes secrete zinc that promotes sympathetic innervation, and administration of zinc ameliorates obesity by promoting sympathetic neuron-induced thermogenesis in mice (202). These studies revealed the beneficial and critical role of sympathetic innervation in maintenance of thermogenic fat in response to cold exposure and other environmental challenge.

## Inter-organ communications around thermogenic fat

**Figure 3:**
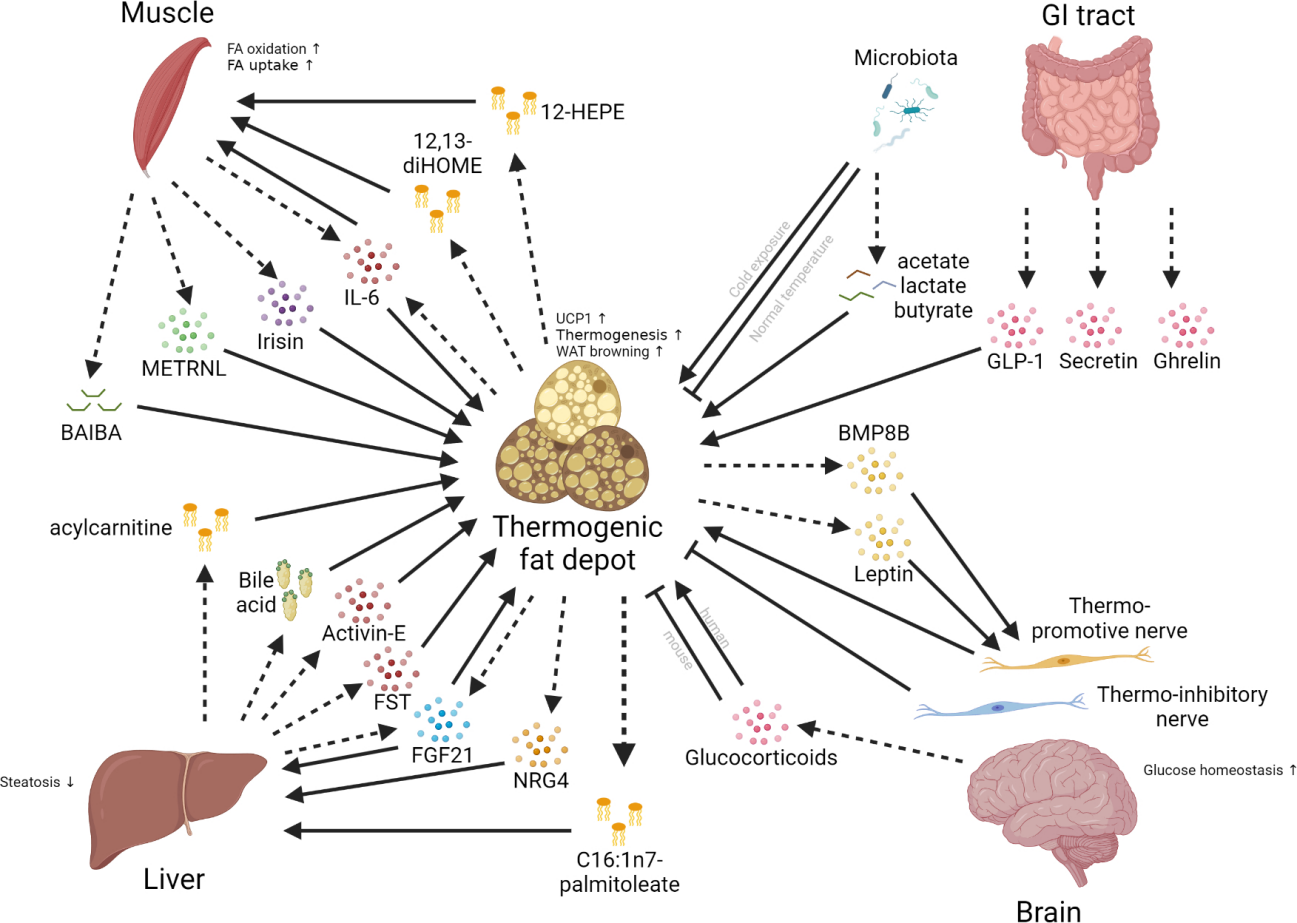
Inter-organ communications between thermogenic fat depots and different organs. Multiple organs, such as the brain, liver, muscle, and gut, can have crosstalk with thermogenic fat depots. The communications between these organs and the thermogenic fat depot mainly involve the secretion of different kinds of molecules, including peptide hormones, lipokines, glucocorticoids, and bile acids. Dashed arrows mean the secretion of factors; solid arrows mean positive effects; blunt-end lines mean inhibitory effects.

The coordination of multiple tissues and organs is very important for maintaining systemic homeostasis and responding to nutritional and environmental challenges, and its dysregulation leads to various metabolic disorders (203–205). The thermogenic fat function as an endocrine organ by secreting specific factors (brown adipokines or batokines) and interact with distant organs that express the corresponding receptors, and *vice versa* (**Figure 3**).

### Brain-thermogenic fat communication

Besides the local effects of nerve on the thermogenic fat, the brain-thermogenic fat communication axis plays an important role in regulating systemic energy balance. Adipose tissue transmits the message to the brain *via* secreted factors and sensory innervation (206, 207). Leptin, an adipokine, is mainly produced by the *obese* (*ob*) gene in adipocytes, and regulates the balance of energy *via* decreasing food intake and inducing energy expenditure (208, 209). Although the role of leptin in regulating energy balance is well known, the underlying mechanism is still elusive. Recent work has shown that leptin target the melanocortin receptor 4 (MC4R) and melanocortin receptor 3 (MC3R) in the brain of mice (210, 211). *Mc4r*-deficient mice exhibit reduced upregulation of *Ucp1* in BAT exposed to cold condition or high-fat food (212). In contrast, central administration of MC3/4-R agonists MTII promote *Ucp1* mRNA expression in mice (213), suggesting the role of MC4R-expressing neuronal populations in regulating BAT thermogenesis. It was also shown that leptin and insulin act synergically on hypothalamic neurons to promote iWAT browning in mice (214). Bone morphogenetic protein 8b (BMP8b), a factor induced by nutritional and thermogenic stimuli in mature BAT and hypothalamus, is also involved in central control of BAT thermogenesis, and central BMP8B treatment increases sympathetic activation of BAT in mice, depending on the hypothalamic AMP-activated protein kinase (AMPK) activation (215).

Central control could also inhibit the browning process, as fasting and chemical-genetic activation of orexigenic agouti-related protein (AgRP) neurons in the hypothalamus suppress iWAT browning in mice (216). Mechanistically, the levels of O-linked β-N-acetylglucosamine (O-GlcNAc) transferase and O-GlcNAc modification in AgRP neurons are increased after fasting in mice, thus promoting neuronal excitability and inhibiting iWAT browning (216). It was also reported that glucocorticoids, a class of steroid hormones synthesized in the adrenal cortex, also suppress *Ucp1* expression and BAT thermogenesis in mice (217). In contrast, the glucocorticoids promote *UCP1* expression in human brown adipocytes and increase glucose uptake and energy expenditure in response to mild cold condition (218). Understanding the species-specific action of glucocorticoid on BAT thermogenesis will provide not only the understanding for BAT-brain axis, but also new therapeutic strategy for maintaining energy homeostasis. Overall, these studies show the differential effects of central control of function of thermogenic fat, mainly depending on the different types of neurons.

### Liver-thermogenic fat communication

The liver is a metabolic organ important for glucose and lipid metabolism, whose dysfunction leads to many kinds of metabolic diseases. The interaction between the liver and thermogenic fat are mainly mediated by peptide hormones, lipokines as well as bile acids. Fibroblast growth factor 21 (FGF21) is a circulating peptide hormone, which is mainly expressed in the liver in response to starvation or exercise and induced in BAT and WAT when fasted or exposed to cold environment both in mice and humans (219). FGF21 not only acts locally in an endocrine and autocrine manner, but also travels to distant organs to exert its role by secreting into the bloodstream (220). Studies showed that administration of FGF21 increases energy expenditure and improves insulin sensitivity in mice (221). Owen et al. further revealed that FGF21 improves energy expenditure through enhanced sympathetic nerve activity in BAT of mice (222). Moreover, the administration of recombinant FGF21 for 6 weeks in diabetic rhesus monkeys lead to a significant decline in glucose level, body weight, and circulating lipids levels (223). Similarly, Activin-E, a member of transforming growth factor beta (TGFβ) superfamily, is primarily produced by the liver and functions as a hepatokine to activate thermogenesis both in iWAT and BAT of mice (224, 225). Follistatin (Fst), which binds and neutralizes the activity of TGFβ superfamily, is secreted by the liver and promotes brown preadipocyte differentiation and cold-induced brown thermogenesis in mice, although the autocrine effect could not be excluded, since *Fst* is also induced in brown adipocytes in response to cold (226–228).

On the other hand, brown adipocytes secrete batokines to regulate the functions of the liver. As discussed above, FGF21 mediate the bi-directional crosstalk between BAT and the liver in mice (204, 221, 222). Besides, brown adipocyte-derived Neuregulin 4 (Nrg4), a member of the epidermal growth factor (EGF) family of ligands, attenuates hepatic lipogenic signaling and protects mice against diet-induced insulin resistance and hepatic steatosis (142). In mice, acute psychological stress induces IL6 secretion from brown adipocytes and then promotes hyperglycemia through hepatic enhanced gluconeogenesis (229). Other reports revealed that some adipokines, such as adiponectin, suppress hepatic injury induced by alcohol intake in mice model (230).

Another class of molecules that mediate the communication between the liver and thermogenic fat are lipokines, which can be secreted both by the adipose tissue and the liver (231, 232). Through quantitative and systemic lipidomic analyses, Cao et al. identified C16:1n7-palmitoleate as an adipose tissue-derived lipid hormone that functions as an important regulator of metabolic homeostasis, such as suppression of hepatosteatosis in mice (231). Similarly, using non-targeted liquid chromatography-mass spectrometry-based lipidomics, Simcox et al. identified that acylcarnitine,produced by the mouse liver in response to cold exposure, transports to BAT to induce UCP1-dependent uncoupling respiration and heat production (232). Bile acids also participate in the communication between the liver and thermogenic fat. TGR5, a G-protein-coupled receptor, could bind to the bile acids transported to brown or beige adipocytes from the liver and induce cold-induced thermogenesis in mice (233–235). BAT also regulate liver inflammation, although the exact pathway governing this crosstalk remains unclear. Previous studies showed that *Ucp1* ^-/-^ mice exhibits decreased capacity to clear succinate from both the liver and the circulation, thus driving liver inflammation through the interaction with stellate cells and macrophages (236, 237). Collectively, these studies show that the intensive crosstalk between the liver and thermogenic fat mediated by various circulating factors, including peptide hormones, lipokines as well as bile acids.

### Skeletal muscle-thermogenic fat communication

Upon muscle contraction, skeletal muscles produce and release circulating cytokines and other peptides, known as myokines, which exert endocrine effects and mediate the communication between muscle and other organs (238–240). In reciprocal, cold- or exercise-induced batokines from thermogenic fat also regulate the function of skeletal muscle.

The earliest identified and most studied myokine is IL-6, which can increase up to 100 folds in circulation during physical exercise (241). Daily injection of IL-6 for 1 week significantly increases *Ucp1* mRNA levels in iWAT of mice (242). Moreover, administration of recombinant human IL-6 enhances lipolysis as well as fatty acid oxidation both in healthy young and elderly humans (243, 244). Consistent with this, elevated IL-6 secretion is also observed in differentiating human beige adipocytes, and blockage of IL-6 receptor by specific antibody inhibits human brown adipocyte differentiation (245). Irisin is another myokine that mediates the communication between skeletal muscle and thermogenic fat, which is secreted from skeletal muscle in a PGC1α-dependent manner and stimulates *Ucp1* expression and thermogenesis both *in vitro* and *in vivo* (246). Irisin is also induced by cold exposure in human and promotes brown fat thermogenesis in collaboration with FGF21, representing a cold-activated endocrine axis regulating both shivering and non-shivering thermogenesis (247). METRNL is released by skeletal muscle and adipose tissue after exercise or upon cold exposure respectively, and significantly promotes browning of WAT depots (183), stimulates energy expenditure and improves glucose tolerance, which is mediated by the recruitment of resident eosinophil in WAT depots of mice (196). Roberts et al. identified β-aminoisobutyric acid (BAIBA), a myokine secreted after exercise, increases the expression of brown adipocyte marker genes and induces a brown adipocyte-like phenotype both in human iPSC-derived white adipocytes and in white adipose depot of mice (248).

Meanwhile, batokines from thermogenic fat also regulate the function of skeletal muscle. 12,13-dihydroxy-9Z-octadecenoic acid (12,13-diHOME), a lipokine secreted from BAT when exposed to cold or exercise in mice and human, increases skeletal muscle fatty acid oxidation and uptake (249, 250). 12-hydroxyeicosapentaenoic acid (12-HEPE), a 12-lipoxygenase-derived lipokine that is secreted in response to cold exposure and β-AR signaling, also promotes glucose uptake in muscle as well as BAT in mice (251). These studies clearly show the mutually regulatory network between skeletal muscle and thermogenic fat to maintain thermogenic fat homeostasis.

### GI tract-thermogenic fat communication

The gastrointestinal tract (GI tract) plays a very important role in thermogenesis through gut microbiota or directly secreting factors from intestinal cells (252). In a study that compared the metabolic profiling between germ-free mice and conventional mice, Mestdagh et al. revealed increased lipolysis while reduced lipogenesis in BAT of germ-free mice (253). Suarez et al. also showed that depletion of microbiota, either by antibiotic treatment or in germ-free mice, promote the browning of iWAT and perigonadal visceral adipose tissue in lean mice, obese mice and high-fat diet-fed mice (254). However, Zietak et al. found cold exposure markedly alter the microbiome composition, and cold-adapted microbiota improved energy metabolism (255). Transplantation of the gut microbiota from cold-induced mice to germ-free mice increase insulin sensitivity, cold tolerance, and browning of WAT (256). Other study revealed that acetate and lactate from the gut microbiota promote the browning of iWAT of mice after intermittent fasting, although the underlying mechanism remains unclear (257). Of note, administration of the bacterial metabolite butyrate also increases the thermogenic capacity of the germ-free mice (258).

Besides gut microbiota, the GI tract also secrete various factors to regulate thermogenesis. Secretin, secreted by the gut and upregulated during fasting, increases lipolysis and inhibits glucose uptake in mice (259). Li et al. revealed that secretin mediates a gut-BAT-brain axis, which stimulates brown fat thermogenesis and satiation in mice (260). The similar role of secretin is also observed in human (261). Glucagon-like peptide 1 (GLP-1), a peptide released from enteroendocrine cells in the gut, increases insulin secretion in beta cells and activates BAT thermogenesis in mice (262). GLP-1 has also been proved to increase satiety and reduce energy intake in human (263). GLP-1 agonists significantly induce BAT thermogenesis and promote browning of iWAT in mice (264). Numerous evidences support that GLP-1 agonists decrease the risk of developing cardiovascular disease in diabetes and obesity both in mice and humans (265). Ghrelin, another growth-hormone-releasing acylated peptide from stomach, also modulates thermogenesis in BAT as well as lipid utilization in WAT, possibly through the gut-brain-BAT axis, as this occurs when ghrelin was centrally administered in mice (266–269). Further studies need to investigate whether and how thermogenic fat could influence the gut homeostasis, as this has not been explored in depth so far.

## Conclusion

Understanding of the development route of thermogenic fat will provides novel therapeutic interventions for metabolic diseases. In this review, we discussed the regulatory network of thermogenic fat at the molecular and cellular levels, respectively. The molecular regulation of thermogenic fat mainly involves transcriptional regulation, epigenetic regulation, non-coding RNA regulation and metabolic reprogramming. Among these regulators, PPARγ, PRDM16 and PGC1α represent the core regulators, as most of the other regulators regulate the thermogenesis depending on them. Besides, thermogenic fat is also educated by other cell types within adipose depots or other organs. These complex and comprehensive regulatory networks help to maintain the functionality of thermogenic fat in response to kinds of changes of the environment. This holds a promising strategy for inducing artificial thermogenesis to counteract obesity *in vivo*. For example, recent study has shown that thermogenesis could be induced through local hyperthermia therapy, mainly through the HSF1-A2B1 transcriptional axis (270). However, whether this kind of induced thermogenesis represents a new specific regulatory network or converges on the core regulators still needs to be identified. In future, more advanced technology, such as spatial transcriptomics and epigenomics methodologies, should be applied to this field to better delineate the development route of thermogenic fat.

## Author contributions

All authors listed have made a substantial, direct, and intellectual contribution to the work and approved it for publication.
